# ^18^F-FDG PET/CT for the quantification of inflammation in large carotid artery plaques

**DOI:** 10.1007/s12350-017-1121-7

**Published:** 2017-12-05

**Authors:** Kjersti Johnsrud, Karolina Skagen, Therese Seierstad, Mona Skjelland, David Russell, Mona-Elisabeth Revheim

**Affiliations:** 1 Division of Radiology and Nuclear Medicine, Oslo University Hospital, Postbox 4950, Nydalen, 0424 Oslo, Norway; 2 Institute of Clinical Medicine, University of Oslo, Postbox 1171, Blindern, 0318 Oslo, Norway; 3 Department of Neurology, Oslo University Hospital, Postbox 4950, Nydalen, 0424 Oslo, Norway

**Keywords:** ^18^F-FDG PET/CT, carotid plaque, atherosclerosis, inflammation, quantification method

## Abstract

**Background:**

There is currently no consensus on the methodology for quantification of ^18^F-FDG uptake in inflammation in atherosclerosis. In this study, we explore different methods for quantification of ^18^F-FDG uptake in carotid atherosclerotic plaques and correlate the uptake values to histological assessments of inflammation.

**Methods and Results:**

Forty-four patients with atherosclerotic stenosis ≥70% of the internal carotid artery underwent ^18^F-FDG PET/CT. Maximum standardized uptake values (SUV_max_) from all plaque-containing slices were collected. SUV_max_ for the single highest and the mean of multiple slices with and without blood background correction (by subtraction (cSUV) or by division (target-to-background ratio (TBR)) were calculated. Following endarterectomy 30 plaques were assessed histologically. The length of the plaques at CT was 6-32 mm. The ^18^F-FDG uptake in the plaques was 1.15-2.66 for uncorrected SUVs, 1.16-3.19 for TBRs, and 0.20-1.79 for cSUVs. There were significant correlations between the different uptake values (*r* = 0.57-0.99, *P* < 0.001). Methods with and without blood background correction showed similar, moderate correlations to the amount of inflammation assessed at histology (*r* = 0.44-0.59, *P* < 0.02).

**Conclusions:**

In large stenotic carotid plaques, ^18^F-FDG uptake reflects the inflammatory status as assessed at histology. Increasing number of PET slices or background correction did not change the correlation.

**Electronic supplementary material:**

The online version of this article (10.1007/s12350-017-1121-7) contains supplementary material, which is available to authorized users.

## Introduction

Inflammation is a key factor in the pathophysiology of atherosclerosis with regard to progression and destabilization of plaques.^1^ Patients with unstable carotid plaques have increased risk of plaque rupture and ischemic stroke,^2^,^3^ and there is increasing interest in imaging carotid plaque inflammation in order to detect these unstable plaques.

Positron emission tomography (PET) imaging with 2-deoxy-2-(^18^F)-fluoro-d-glucose (^18^F-FDG) of inflammation in atherosclerotic plaques has rapidly evolved since Rudd et al first reported ^18^F-FDG accumulation in macrophage-rich areas of carotid artery plaques over a decade ago.^4^ In contrast to oncology,^5^ there is no consensus on methodological guidelines for ^18^F-FDG PET/CT in atherosclerosis imaging. A recent position paper from the Cardiovascular Committee of the European Association of Nuclear Medicine^6^ proposed optimized and standardized protocols for the imaging and interpretation of ^18^F-FDG PET scans in atherosclerosis. However, they admitted that many of the recommendations suffer from the absence of conclusive evidence. Compared to a solid tumor, the cells responsible for ^18^F-FDG uptake in carotid artery plaques are generally fewer, more dispersed, and spread around parts of the circumference of a tubular vascular structure.^3^,^7^ Consequently, limited spatial resolution of the PET scanner and blood background activity are of great concern. Two parallel phenomena are known to influence measured activity in a lesion:^8^ signal from the lesion lost to the surroundings (spill-out), and signal added to the lesion from the vessel lumen and adjacent anatomic structures (spill-in).^8^,^9^

Different acquisition protocols and quantification methods have been suggested.^10^-^17^ They all address the same concerns but have diverging solutions. A literature search identified 53 different acquisition protocols, 51 different reconstruction protocols, and 46 different quantification methods used in 49 studies.^9^ The most common measure is the mean of all the maximum standardized uptake values (SUVs) (mean SUV_max_) of the regions of interest (ROIs). The ROIs include the whole plaque (in localized stenosis), or one or more vessel segments (in subclinical/generalized disease). Bural et al calculated the atherosclerotic burden of the aorta by multiplying the mean uptake values for each aorta segment with the vessel wall volume.^18^ Sub-analysis looking for the most metabolically active areas of a vessel segment have been used in therapy response studies.^16^ The uptake values are either normalized to the blood background activity,^15^,^17^,^19^,^20^ corrected for the background activity with subtraction^11^ or not corrected for background activity.^13^,^21^,^22^ The rationale for background correction has been strongly criticized.^9^,^23^

The aim of this study was to explore different methods for the quantification of ^18^F-FDG uptake in large carotid artery plaques, and to correlate the uptake values to the amount of inflammation on histology.

## Methods

### Study Population

Forty-four patients referred to the Department of Neurology at our institution for the evaluation of carotid artery disease were included (Table 1). Inclusion criteria were ultrasound-confirmed atherosclerosis with internal carotid artery stenosis ≥ 70% according to consensus criteria of the Society of Radiologists in Ultrasound.^24^ Exclusion criteria were prior carotid endarterectomy/angioplasty with stenting, carotid occlusion, vasculitis, malignancy, prior radiation therapy to the neck, or immunotherapy. The Regional Committee for Medical and Health Research Ethics approved the study and all patients provided informed written consent.

**Table 1:** Patient characteristics (*n* = 44)

Age, years; mean ± SD	66.3±8.4
Sex, male; *n* (%)	30 (68.2)
Statin therapy; *n* (%)	34 (77.3)
Blood glucose, mmol L^−1^; mean ± SD (range)	6.8 ± 2.2 (4.9-14.9)
Bodyweight, kg; mean ± SD (range)	83.5 ± 16 (55-110)
Body mass index, kg/m^2^; mean ± SD (range)	27.4 ± 4.5 (19.9-34.8)

A subgroup of the patient material is included in a previously published study.^25^

Of the 44 included patients, 38 underwent carotid endarterectomy due to ipsilateral ischemic events or as prophylactic treatment before heart surgery. Eight of these plaques were lost to histological assessment due to logistical reasons.

### ^18^F-FDG PET/CT Examination

Following overnight fasting blood glucose level was measured before the patient received *i.v.* injection of 5 MBq/kg (0.14 mCi/kg). After a mean circulation time of 100 minutes (range 68-156), a ^18^F-FDG PET/CT scan from the base of the skull to the aortic arch was performed with 15 minutes per bed position using a hybrid PET/CT scanner (Siemens Biograph 64, Siemens Medical Systems, Erlangen, Germany). The PET data were reconstructed to 2-mm slices with a pixel size of 2.67 mm using the ordered subset expectation-maximization 2D algorithm with four iterations (i), eight subsets (s)(4i/8s), and Gaussian post-reconstruction filter with full-width at half maximum of 3.5 mm. For attenuation correction, low-dose CT without the use of *i.v*. contrast was performed immediately before ^18^F-FDG PET. For patients without a recent CT angiography of the carotid arteries, this was performed after ^18^F-FDG PET.

### ^18^F-FDG Uptake Quantification

**Figure 1:**
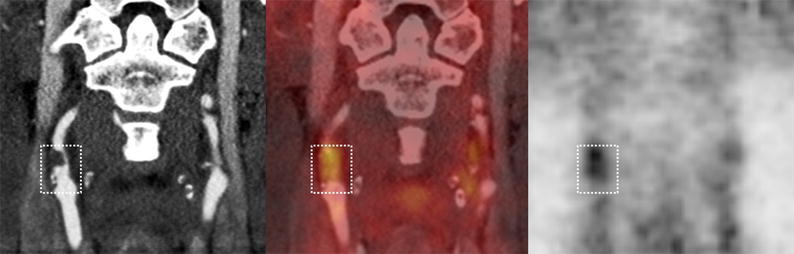
^18^F-FDG PET/CT of stenotic plaque in the right internal carotid artery. From left to right: CT angiography, co-registered PET/CT, and ^18^F-FDG PET. The white box shows the plaque extension craniocaudally with lumen contrast-filling defect and vessel wall thickening on CT angiography (left image)

**Figure 2:**
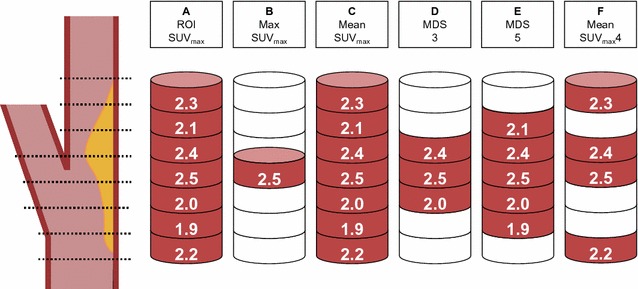
^18^F-FDG uptake quantification was based on the SUV_max_ of all the plaque-containing axial PET slices (**A**): Max SUV_max_ = the single highest SUV_max_ (**B**), mean SUV_max_ = the mean of all plaque SUV_max_ (**C**), MDS3 = the mean SUV_max_ of the three contiguous slices centered on the slice with the highest SUV_max_ (**D**), MDS5 = the mean SUV_max_ of the five contiguous slices centered on the slice with the highest SUV_max_ (**E**), and mean SUV_max_4 = the mean SUV_max_ of the four slices with highest SUV_max_ within the plaque(F) (based on a figure by Tawakol et al ^32^)

The Hybrid Viewer 2.0 software (Hermes Medical Solutions AB, Stockholm, Sweden) was used for image fusion and ^18^F-FDG uptake quantification. The CT angiography was used to guide drawing of the ROIs on the fused PET/CT slices. A plaque was defined as vessel wall thickening and a lumen contrast-filling defect on CT angiography (Figure 1).^13^ An experienced nuclear medicine physician (K.J.) drew ROIs around the entire vessel wall and lumen on all plaque-containing axial PET slices (Figure 2). ROIs were carefully placed to minimize the influence from ^18^F-FDG uptake in structures close to the plaque (e.g., lymph nodes, paravertebral muscles, or salivary glands). Blood pool activity was obtained from four ROIs placed in the lumen of the jugular vein away from structures with ^18^F-FDG uptake. Plaque localization in relation to the carotid bifurcation was recorded. The most cranial slice of the common carotid artery before the division was defined as the bifurcation. The pixel values in the PET images were converted into SUV normalized to lean body mass.^5^

For all plaque ROIs, SUV_max_ was obtained and the ^18^F-FDG uptake was quantified using the following approaches (Figure 2):

1. Max SUV_max_ = the single highest SUV,
2. Mean SUV_max_ = the mean of all plaque SUV_max_,
3. Most Diseased Segment (MDS)3 = the mean SUV_max_ of the three contiguous slices centered on the slice with the highest SUV_max_,
4. MDS5 = the mean SUV_max_ of the five contiguous slices centered on the slice with the highest SUV_max_,
5. Mean SUV_max_4 = the mean SUV_max_ of the four slices with highest SUV_max_.

For all blood pool ROIs, SUV_mean_ was obtained. Blood background-corrected values for all the SUV measurements were calculated (target-to-background ratio (TBR); SUV divided by the blood pool activity (mean SUV_mean_ in four venous regions) and corrected SUV (cSUV); blood pool-corrected SUV (subtraction of the blood pool activity (mean SUV_mean_) from SUV).

A second independent experienced nuclear medicine physician (MER) drew ROIs on the 20 initial patients to assess inter-observer variability of the different quantification methods.

### Endarterectomy and Histological Analysis

**Figure 3:**
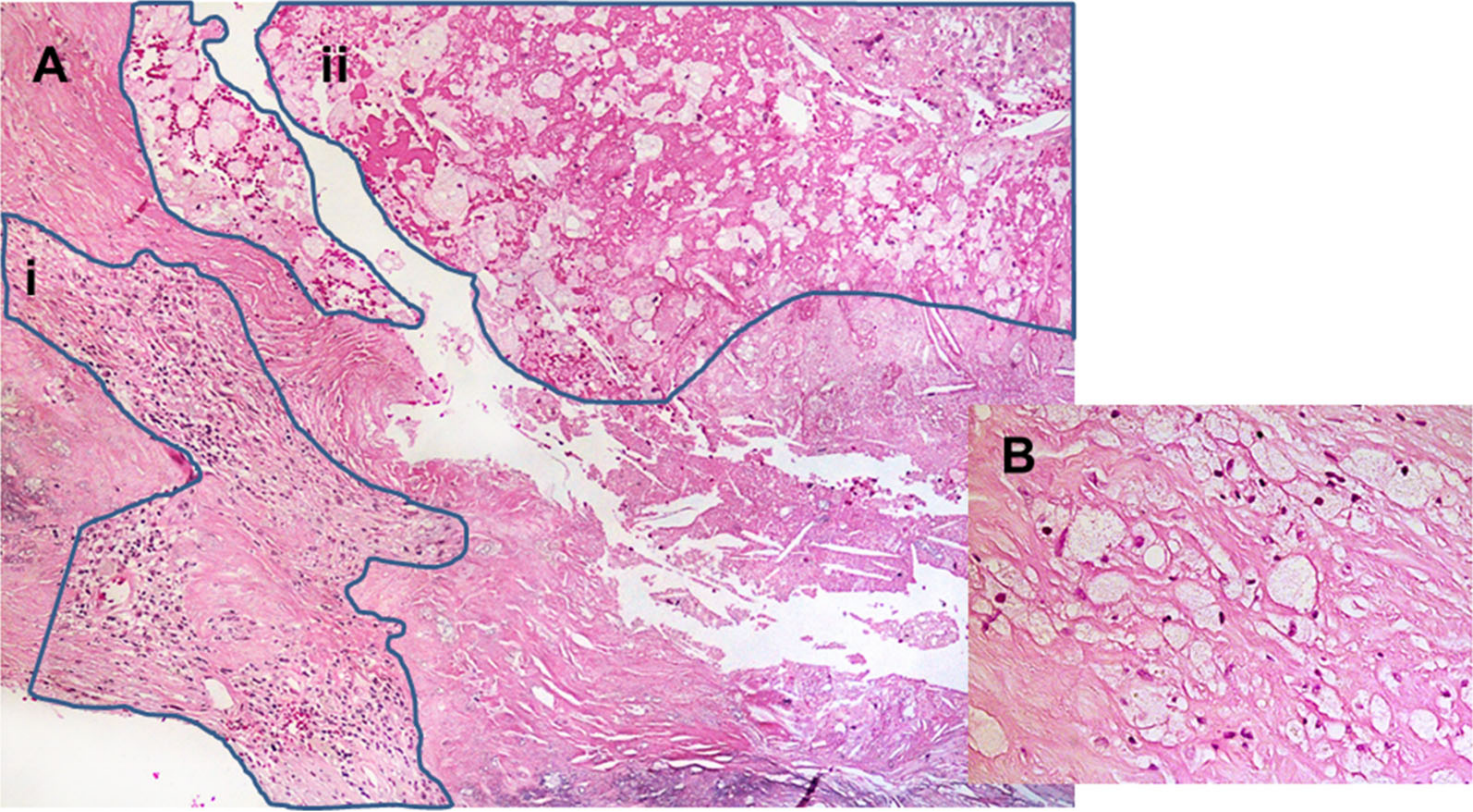
The histological quantification of inflammation was performed on H&E stained samples. The ocular micrometer was used to measure the total plaque area and area occupied by inflammatory cells in all the fields of view from all sections. The amount of inflammation per plaque was defined as the sum of all areas with inflammatory cells divided by the total area of all sections. **A**: ×200 magnification with inflammatory areas (marked by blue lines) containing mainly lymphocytes (*i*) and lipid macrophages (*ii*). **B**: ×400 magnification of areas with lipid macrophages

The mean time between PET/CT and endarterectomy was 6 days (range; 0-116, median; 0). The histological analysis has been described previously.^25^ In brief, plaques were removed *en bloc* at carotid endarterectomy, fixed in 4% formaldehyde, decalcified in ethylenediaminetetraacetic acid, and cut into 2- to 3-mm slices. After dehydration, the slices were embedded in paraffin and a 5-µm histological section from each slice was cut and stained with hematoxylin and eosin (H&E). The number of histology sections obtained from each plaque ranged from 2 to 9 (mean 4.9, SD 2.0). The plaques were assessed by a pathologist and a research physician blinded for the clinical and the ^18^F-FDG PET findings. Inflammation was quantified using a modified version of the method used by Jander et al.^3^ The sections were evaluated with 120× magnification and the percentage area of inflammatory cells (macrophages and leucocytes) per plaque was obtained (Figure 3): For each section, the total area and the area occupied by inflammatory cells were measured manually. The amount of inflammation per plaque was defined as the sum of all areas with inflammatory cells divided by the total area of all the sections. We performed repeated histopathological assessment on selected sections from this study cohort and found that the amount of inflammatory cells was in the same 5% category at both assessments for 73% of the sections (Kappa = 0.73).^25^ The amount of inflammation per plaque was 6.8% (SD 4.0; range 0.4-17.9).

### Statistical Analysis

The SPSS Statistics software for Windows (IBM, version 21.0; SPSS Inc., Chicago, Ill) was used. Groups of data were compared using two-sided *t*-test and Pearson correlation for normally distributed variables. For non-normal distributions Mann-Whitney test and Spearman correlation were used. Statistical significance was set to 0.05.

## Results

### Localization of Plaque and Highest ^18^F-FDG Uptake

**Figure 4:**
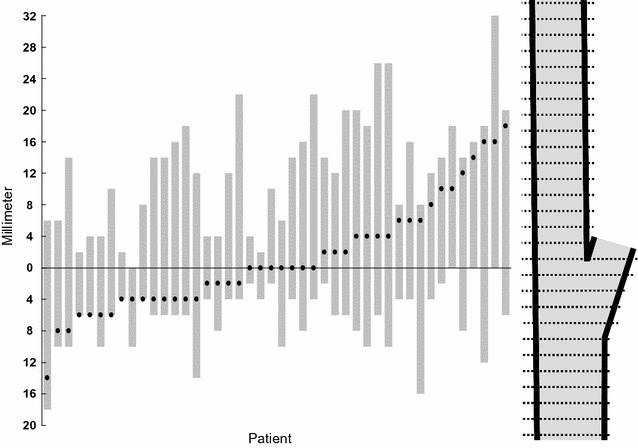
Plaque extension (gray bars) and max SUV_max_ location (black dots) per patient along the *x*-axis. The *y*-axis shows the distance in millimeter from the bifurcation (0) cranially in the internal carotid artery and caudally in the common carotid artery

The length of the plaques in the cranio-caudal direction was 6-32 mm (mean 19, SD 7.6) and all included the carotid artery bifurcation (Figure 4). Max SUV_max_ was located between 14 mm below to 18 mm above the bifurcation (mean 1.2 mm below the bifurcation) (Figure 4).

### Inter-observer Variability

The correlation coefficients between ^18^F-FDG uptakes calculated from plaques delineated by two nuclear medicine physicians independently were 0.96-0.98 for uncorrected SUVs, 0.63-0.68 for TBRs, and 0.90-0.93 for cSUVs. The correlation coefficient for background blood pool activity was 0.75.

### Quantification of ^18^F-FDG Uptake

Mean values, SDs, and ranges for the different quantification methods are summarized in Table 2. The ^18^F-FDG uptake was significantly different for the different quantification approaches (paired samples *t*-test, *P* < 0.004 for all pairs). TBR gave the highest mean values and the widest ranges, whereas the background-subtracted values (cSUV) showed the lowest mean values and the narrowest ranges.

**Table 2:** Plaque SUV for different quantification methods (*n* = 44)

	Uncorrected	TBR	cSUV
Max SUV_max_	1.76 ± 0.35 (1.18-2.66)	2.07 ± 0.44 (1.34-3.19)	0.90 ± 0.33 (0.42-1.79)
Mean SUV_max_	1.56 ± 0.28 (1.11-2.28)	1.83 ± 0.38 (1.16-2.91)	0.69 ± 0.28 (0.20-1.29)
MDS3	1.70 ± 0.34 (1.17-2.51)	2.00 ± 0.43 (1.26-3.16)	0.83 ± 0.32 (0.33-1.64)
MDS5	1.66 ± 0.32 (1.15-2.32)	1.95 ± 0.41 (1.22-3.14)	0.79 ± 0.31 (0.28-1.45)
Mean SUV_max_4	1.68 ± 0.33 (1.15-2.45)	1.98 ± 0.42 (1.26-3.16)	0.82 ± 0.31 (0.32-1.58)

Data are given as mean ± SD (range)

*TBR*, target-to-background ratio; *SUV*, standardized uptake value; *cSUV*, corrected SUV (background-subtracted SUV); *MDS*, most diseased segment

**Figure 5:**
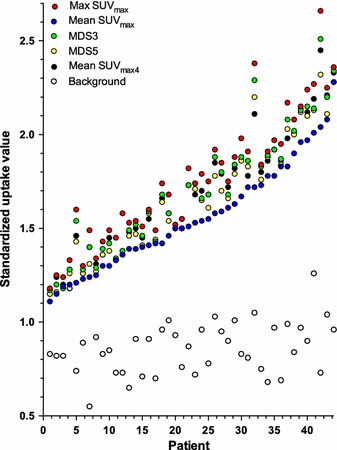
Uncorrected ^18^F-FDG uptake values for individual patients sorted according to increasing mean SUV_max_. Each color represents separate quantification methods. The black dots are the background values (mean SUV_mean_)

**Figure 6:**
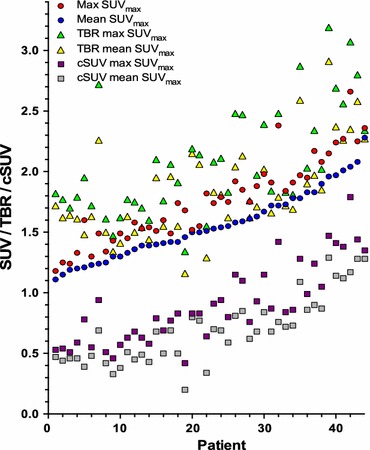
Uncorrected and background-corrected (TBR and cSUV) max and mean SUV_max_ per patient sorted according to increasing mean SUV_max_

^18^F-FDG uptake for the different quantification methods and corresponding background values for all patients are shown in Figure 5. Mean difference between max SUV_max_ and mean SUV_max_ was 0.08 and ranged from 0.02 to 0.66. The effect of background correction is shown in Figure 6. Correlations between ^18^F-FDG uptake within the groups were 0.93-0.99 for the uncorrected SUV, 0.94-1.0 for TBR, and 0.92-0.99 for cSUV (Table 3).

**Table 3:** Correlation coefficients between different methods for ^18^F-FDG uptake quantification (*n* = 44)

	Uncorrected				TBR					cSUV				
	Mean SUV_max_	MDS3	MDS5	Mean SUV_max_4	Max SUV_max_	Mean SUV_max_	MDS3	MDS5	Mean SUV_max_4	Max SUV_max_	Mean SUV_max_	MDS3	MDS5	Mean SUV_max_4
Uncorrected														
Max SUV_max_	.93*	.99*	.99*	.99*	.68*	.57*	.68*	.67*	.65*	.91*	.79*	.89*	.89*	.88*
Mean SUV_max_	1	.96*	.96*	.96*	.64*	.65*	.65*	.66*	.65*	.85*	.87*	.86*	.87*	.86*
MDS3		1	.99*	.99*	.69*	.59*	.69*	.68*	.67*	.91*	.81*	.91*	.90*	.89*
MDS5			1	.99*	.67*	.59*	.67*	.68*	.65*	.89*	.81*	.89*	.90*	.88*
Mean SUV_max_4				1	.69*	.62*	.69*	.69*	.68*	.91*	.83*	.90*	.90*	.90*
TBR														
Max SUV_max_					1	.94*	.99*	.99*	.99*	.91*	.89*	.92*	.91*	.92*
Mean SUV_max_						1	.96*	.97*	.97*	.82*	.93*	.85*	.86*	.87*
MDS3							1	1.0*	1.0*	.91*	.90*	.92*	.92*	.93*
MDS5								1	.99*	.90*	.91*	.91*	.92*	.92*
Mean SUV_max_4									1	.89*	.90*	.90*	.90*	.92*
cSUV														
Max SUV_max_										1	.92*	.99*	.99*	.99*
Mean SUV_max_											1	.94*	.95*	.96*
MDS3												1	.99*	.99*
MDS5													1	.99*
Mean SUV_max_4														1

*SUV*, standardized uptake value; *MDS*, most diseased segment, background-corrected data; *TBR*, target-to-background ratio; *cSUV*, background-subtracted SUV

*Correlation is significant at the 0.01 level

### Histology and ^18^F-FDG Uptake

**Figure 7:**
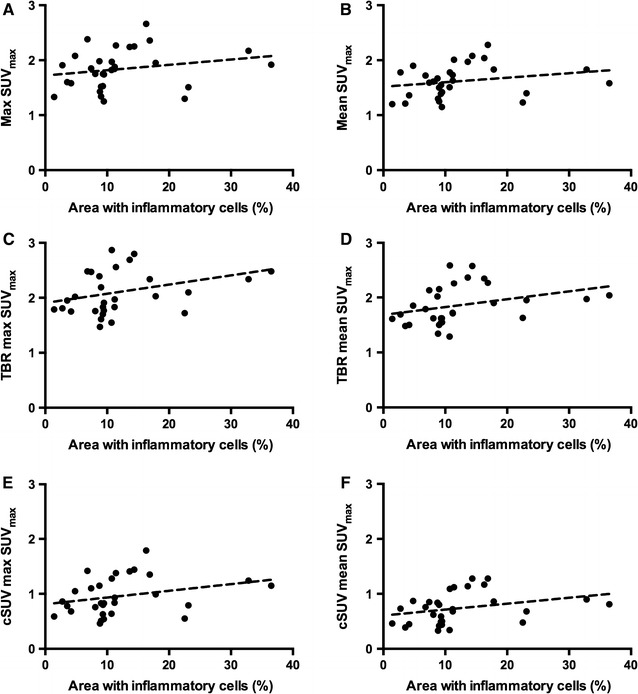
Scatter plots with correlation lines of total area with inflammatory cells versus uncorrected (**A**, **B**) and background-corrected (TBR: **C**, **D** and cSUV: **E**, **F**) max and mean SUV_max_

There were significant moderate (0.44-0.59) correlations between the amount of inflammation and all the different ^18^F-FDG quantification methods (Table 4). The highest correlations were found for mean SUV_max_, and the lowest for max SUV_max_ independent of background correction. Figure 7 shows scatter plots of inflammation versus max and mean SUV_max_ with and without background correction.

**Table 4:** Correlation between ^18^F-FDG PET uptake values and histology (*n* = 30)

^18^F-FDG quantification method	Spearman correlation coefficient
Max SUV_max_	0.48 (0.008)
Mean SUV_max_	0.54 (0.002)
MDS3	0.48 (0.007)
MDS5	0.49 (0.006)
Mean SUV_max_4	0.52 (0.003)
TBR max SUV_max_	0.44 (0.016)
TBR mean SUV_max_	0.58 (0.001)
TBR MDS3	0.47 (0.009)
TBR MDS5	0.48 (0.008)
TBR mean SUV_max_4	0.48 (0.007)
cSUV max SUV_max_	0.47 (0.009)
cSUV mean SUV_max_	0.59 (0.001)
cSUV MDS3	0.52 (0.004)
cSUV MDS5	0.52 (0.003)
cSUV mean SUV_max_4	0.54 (0.002)

Data given as correlation coefficient (*P* value)

## Discussion

In this clinical study, we explored different methods for the quantification of ^18^F-FDG uptake in carotid plaques causing artery stenosis equal to or above 70%. ^18^F-FDG uptake was homogenously disseminated throughout the entire plaques. Although there were differences in magnitude, quantification of ^18^F-FDG uptake with and without background correction showed similar, moderate correlations to inflammation on histology.

**Figure 8:**
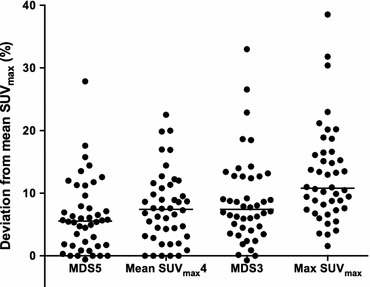
Deviation from the mean SUV_max_ for different quantification methods for individual patients. The black bar represents the mean values

Mean max SUV_max_ (mean of the max SUV_max_ for the study population) was only 13% higher than mean mean SUV_max_ (Figure 8), whereas the three other quantification methods gave values in between, increasing with decreasing number of included slices. Homogenous ^18^F-FDG uptake throughout the plaque contrasts findings from microPET of endarterectomized plaques showing patchy ^18^F-FDG uptake.^26^ The presence of macrophages reduces the thickness of the fibrous capsule and therefore an increasing number is likely to correlate to increasing vulnerability. As such, the highest ^18^F-FDG uptake within a plaque could be the most appropriate parameter for risk assessment. Currently, the most used measure for assessment of plaque inflammation has been the mean SUV_max_.^17^,^21^,^22^ No clinical studies have assessed the use of whole plaque max SUV_max_. Max SUV_max_ is easily obtained, highly reproducible, and less influenced by partial-volume effects.^8^,^27^ Although max SUV_max_ is prone to image noise,^8^ our findings suggest that for atherosclerotic plaque assessment in clinical PET, max SUV_max_ should be explored further.

The strong correlation between the different quantification methods suggests that for this group of patients uncorrected ^18^F-FDG uptake values may provide similar information as background-corrected values. As such, our findings do not support the superiority of TBR as quantification method as suggested by others.^4^,^10^,^14^-^17^ For circulation times above one hour, the blood background is low, but highly variable, whereas carotid plaques have consistent uptake over time.^11^,^13^ A slight variation in blood background will therefore give significant variability in TBR. From a physics perspective, Huet et al 9 have explained that there is no legitimate rationale for using TBR instead of SUV because blood contamination is an additive and not a multiplicative process. A purely additive process would require subtraction of the mean luminal blood pool activity from the SUVs (cSUVs).

The inter-observer variability analysis of ^18^F-FDG quantification revealed superior reproducibility of uncorrected SUV_max_ compared to blood background-corrected SUVs (TBR and cSUV). SUV measurements are highly dependent on the size, shape, and location of the drawn ROI because the ROI can either miss the voxel with the highest intensity or the ROI can inadvertently include contribution from adjacent ^18^F-FDG-avid organs. The use of contrast-enhanced CT to localize the plaques is likely to have contributed to the low inter-observer variability in the SUV_max_ in our study. Our correlation coefficient for blood background of 0.75 inevitably increases the inter-observer variability of all background-corrected values. Other ^18^F-FDG uptake reproducibility studies in localized carotid artery plaques have reported moderate (background corrected)^28^ to excellent (with and without background correction)^22^,^29^ inter-observer agreement. The slightly inferior inter-observer agreement for background-corrected values found in our study could be related to differences in the placement of the ROI in the jugular vein as the diameter of the jugular vein often was small, making the measurement susceptible to image noise. However, this is an inherent limitation of all quantification methods with background correction.

The optimal method should predict plaque vulnerability and clinical outcome. In the present study, all quantification methods for ^18^F-FDG uptake showed moderate correlation to inflammation on histopathology (Table 4). The correlations were systematically slightly higher when mean SUV_max_ was used instead of max SUV_max_. This was not unexpected as total plaque inflammation score and mean SUV_max_ are both multi-slice methodologies. Total plaque inflammation score is a well-established method to correlate histology to ischemic symptoms,^2^,^3^ or to ^18^F-FDG uptake.^17^ When comparing max SUV_max_ with the slice with the highest percentage inflammatory area, the correlation did not increase (data not shown). This is in accordance with Tawakol et al^17^ who found slightly higher correlations between overall plaque inflammation and histology than a slice-by-slice comparison of uptake value and histology. How to use histology as a gold standard to imaging is challenging. We know that excised plaques both shrink^17^ and may be partly damaged.^17^,^25^

The strength of our study was the close timing between the PET examinations and the endarterectomies. Our study patients had plaques not only presumed highly inflammatory giving recent symptoms, but also plaques removed prophylactically from asymptomatic patients.

A limitation of our study is the inclusion of patients with elevated blood glucose. Four had blood glucose values > 11 mmol/L (198 mg/dL) and three 7-11 mmol/L (126-198 mg/dL) at the time of the ^18^F-FDG injection. Elevated blood glucose is known to reduce the uptake of ^18^F-FDG into metabolic active cells in malignant diseases.^30^ Guidelines for the clinical use of ^18^F-FDG in inflammation and infection^31^ also recommend the reduction of blood glucose to the lowest possible level. In studies on atherosclerosis, there is no consensus on a cut-off value.^12^ Some studies do not report on blood glucose level, whereas in other studies the cut-off values have ranged from 8 mmol/L (144 mg/dL)^21^ to 11.1 mmol/L (200 mg/dL).^16^ Our correlations between histology and the different ^18^F-FDG quantification methods were slightly increased when excluding the four patients with blood glucose level > 11 mmol/L. We have not excluded patients with high blood glucose in the correlation analysis of the different uptake parameters and thereby we do not know if this has contaminated our results. In the 44 included patients, there was no correlation between blood glucose and background SUV_mean_. Another limitation is the wide range of circulation times (68-156 minutes) that could have influenced the plaque ^18^F-FDG uptake by underestimating background-corrected values for patients with shorter circulation times and by overestimating background-corrected values for patients with longer circulation times. However, for 38 of the 44 patients the PET acquisition started between 85 and 115 minutes after ^18^F-FDG injection, and thus, it is unlikely that difference in circulation time would change the findings in our study.

## New Knowledge Gained

Our study showed that SUVs without background correction from large plaques in the carotid artery can be used as inflammatory parameter in atherosclerosis.

## Conclusion

In conclusion, in carotid artery stenosis equal to or above 70%, ^18^F-FDG uptake reflects the inflammatory status as assessed on histology. Increasing number of PET slices or background correction did not improve the correlation.

## Electronic Supplementary Material

Below is the link to the electronic supplementary material.

Supplementary material 1 (PPTX 181 kb)

## Abbreviations

PET: Positron emission tomography
^18^F-FDG: 2-deoxy-2-(^18^F)-fluoro-d-glucose
SUV_max_: Maximum standardized uptake value
ROI: Region of interest
MDS: Most diseased segment
TBR: Target-to-background ratio
cSUV: Corrected SUV
