# Supplementary material for: 18F-FDG PET/CT for the quantification of inflammation in large carotid artery plaques
Source: J Nucl Cardiol. 2017 Dec 5;26(3):883–93. doi: 10.1007/s12350-017-1121-7 (PMC6517604; doi:10.1007/s12350-017-1121-7)
Supplement: Supplementary file 1 — Supplementary material 1 (PPTX 181 kb) [file 12350_2017_1121_MOESM1_ESM.pptx]

## Slide 1
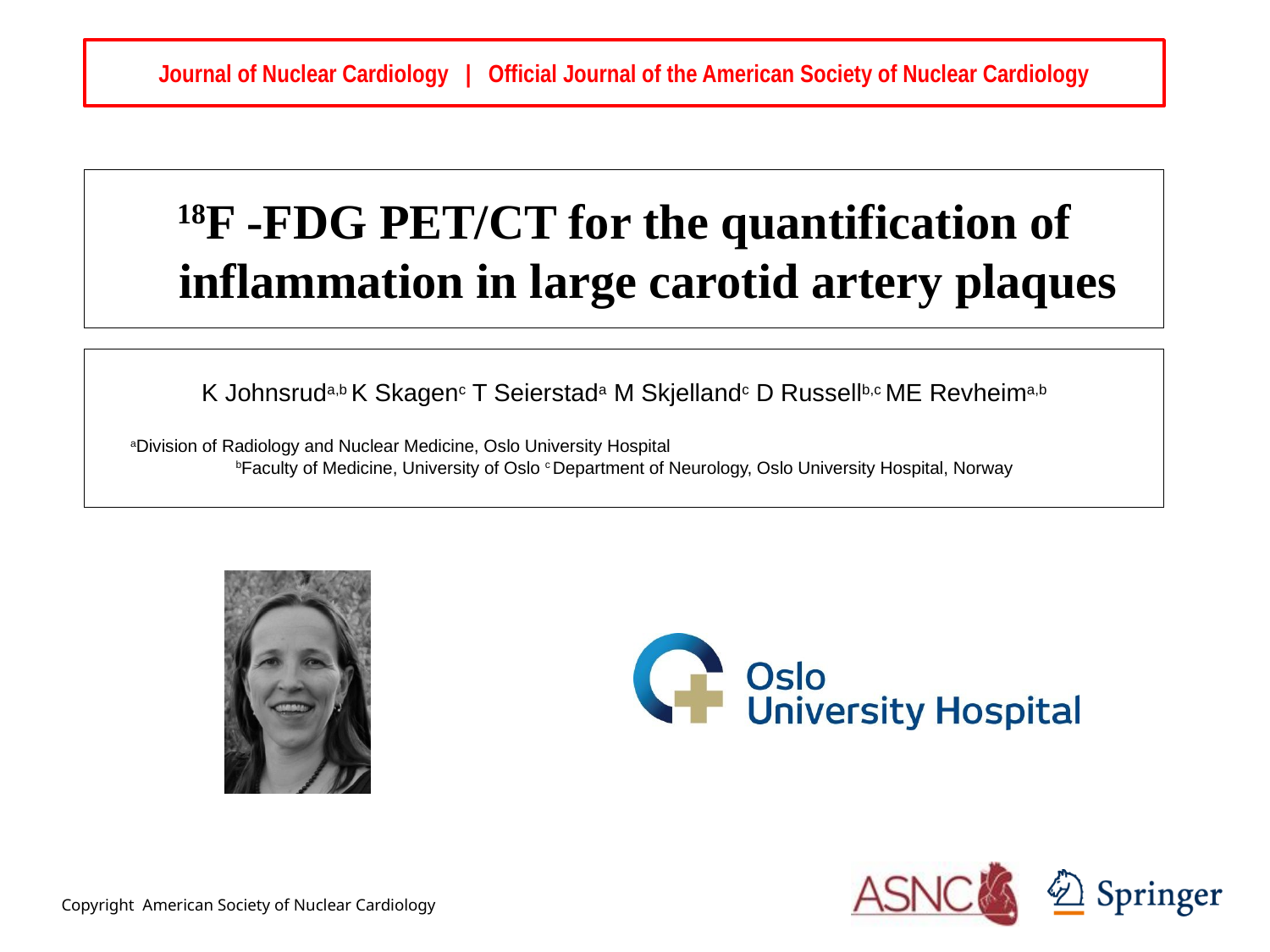

Journal of Nuclear Cardiology | Official Journal of the American Society of Nuclear Cardiology
# 18F -FDG PET/CT for the quantification of inflammation in large carotid artery plaques
K Johnsruda,b K Skagenc T Seierstada M Skjellandc D Russellb,c ME Revheima,b
aDivision of Radiology and Nuclear Medicine, Oslo University Hospital bFaculty of Medicine, University of Oslo c Department of Neurology, Oslo University Hospital, Norway
Copyright American Society of Nuclear Cardiology

## Slide 2
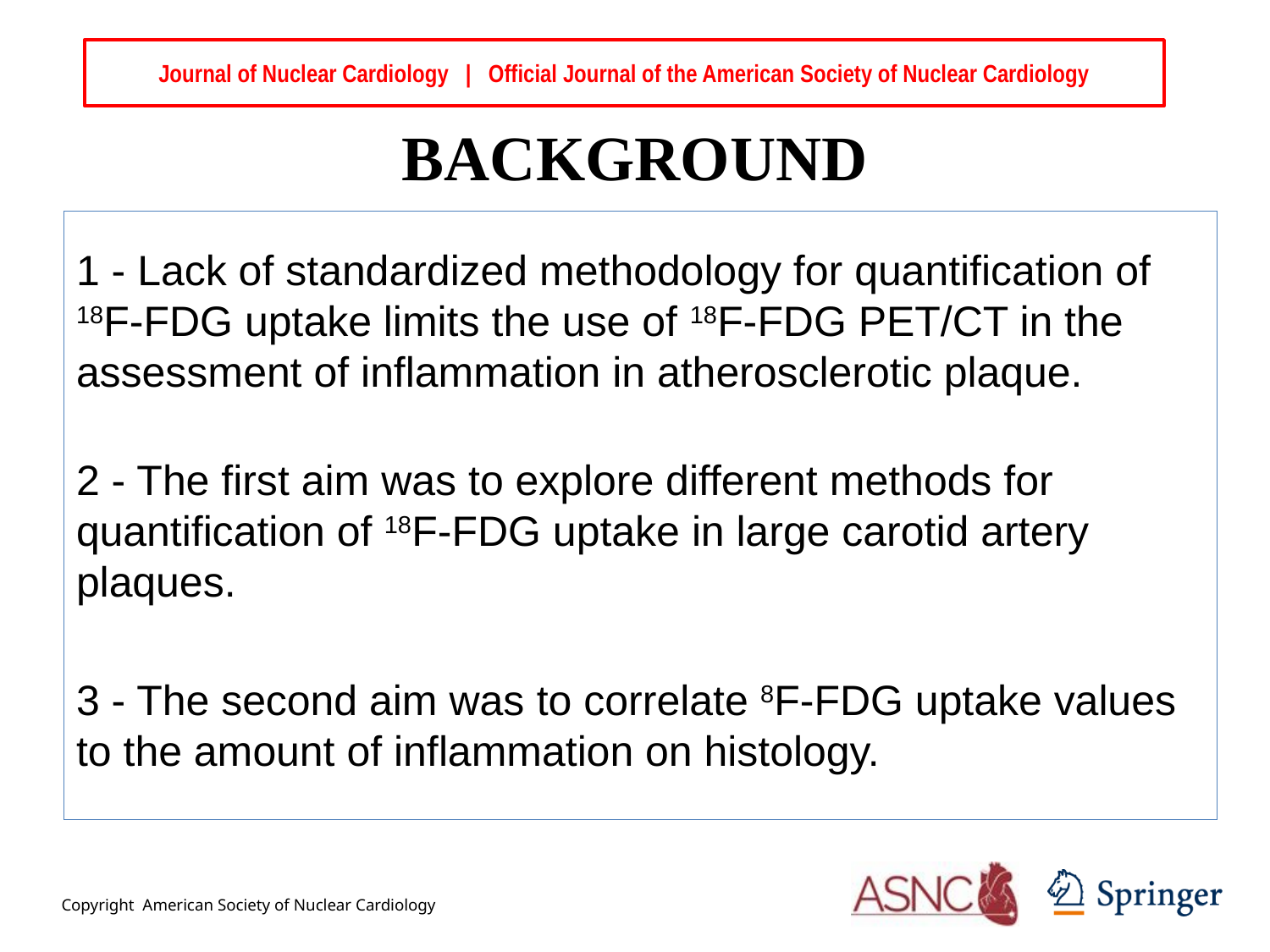

Journal of Nuclear Cardiology | Official Journal of the American Society of Nuclear Cardiology
# BACKGROUND
1 - Lack of standardized methodology for quantification of 18F-FDG uptake limits the use of 18F-FDG PET/CT in the assessment of inflammation in atherosclerotic plaque.
2 - The first aim was to explore different methods for quantification of 18F-FDG uptake in large carotid artery plaques.
3 - The second aim was to correlate 8F-FDG uptake values to the amount of inflammation on histology.
Copyright American Society of Nuclear Cardiology

## Slide 3
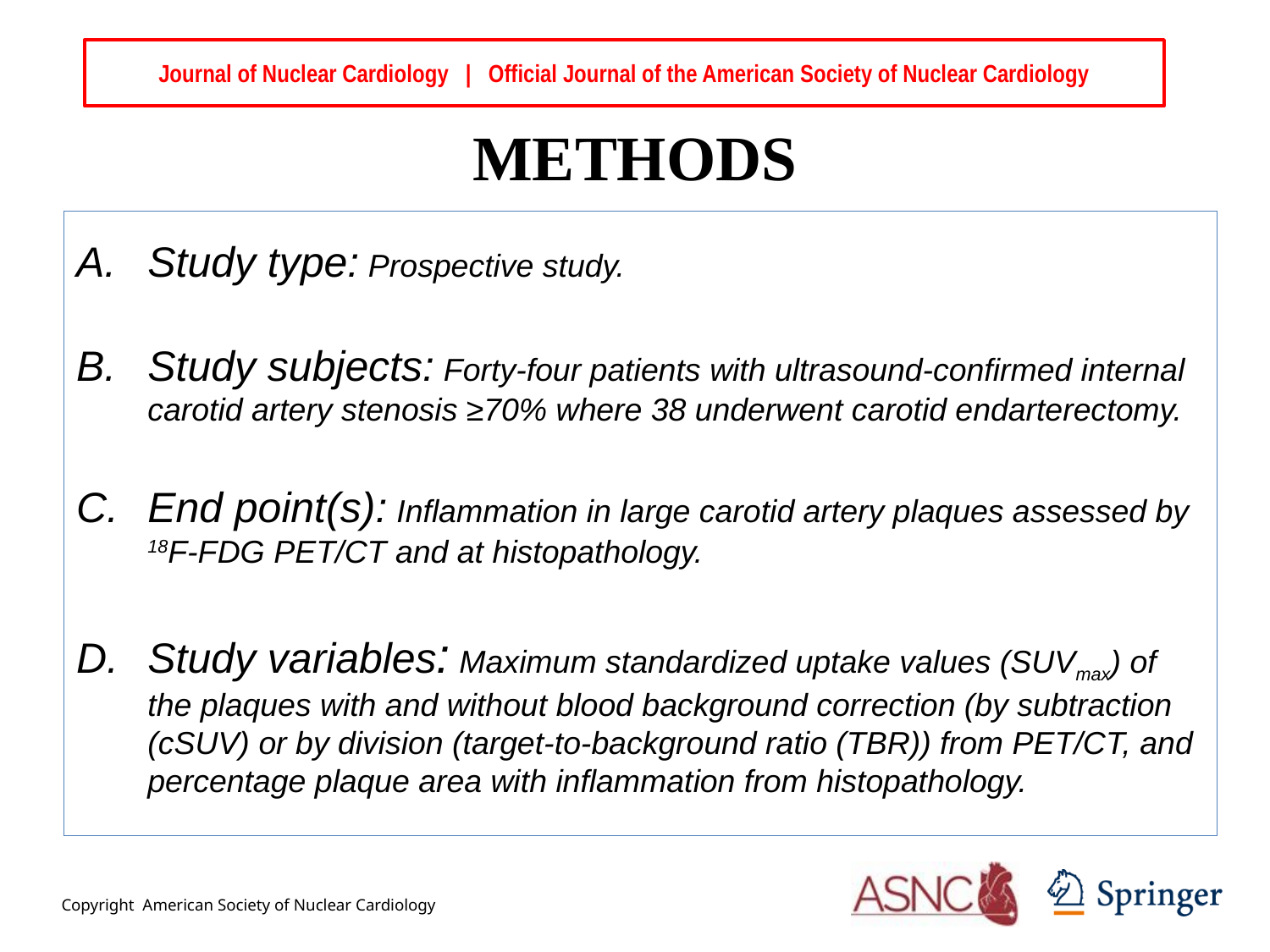

Journal of Nuclear Cardiology | Official Journal of the American Society of Nuclear Cardiology
# METHODS
Study type: Prospective study.
Study subjects: Forty-four patients with ultrasound-confirmed internal carotid artery stenosis ≥70% where 38 underwent carotid endarterectomy.
End point(s): Inflammation in large carotid artery plaques assessed by 18F-FDG PET/CT and at histopathology.
Study variables: Maximum standardized uptake values (SUVmax) of the plaques with and without blood background correction (by subtraction (cSUV) or by division (target-to-background ratio (TBR)) from PET/CT, and percentage plaque area with inflammation from histopathology.
Copyright American Society of Nuclear Cardiology

## Slide 4
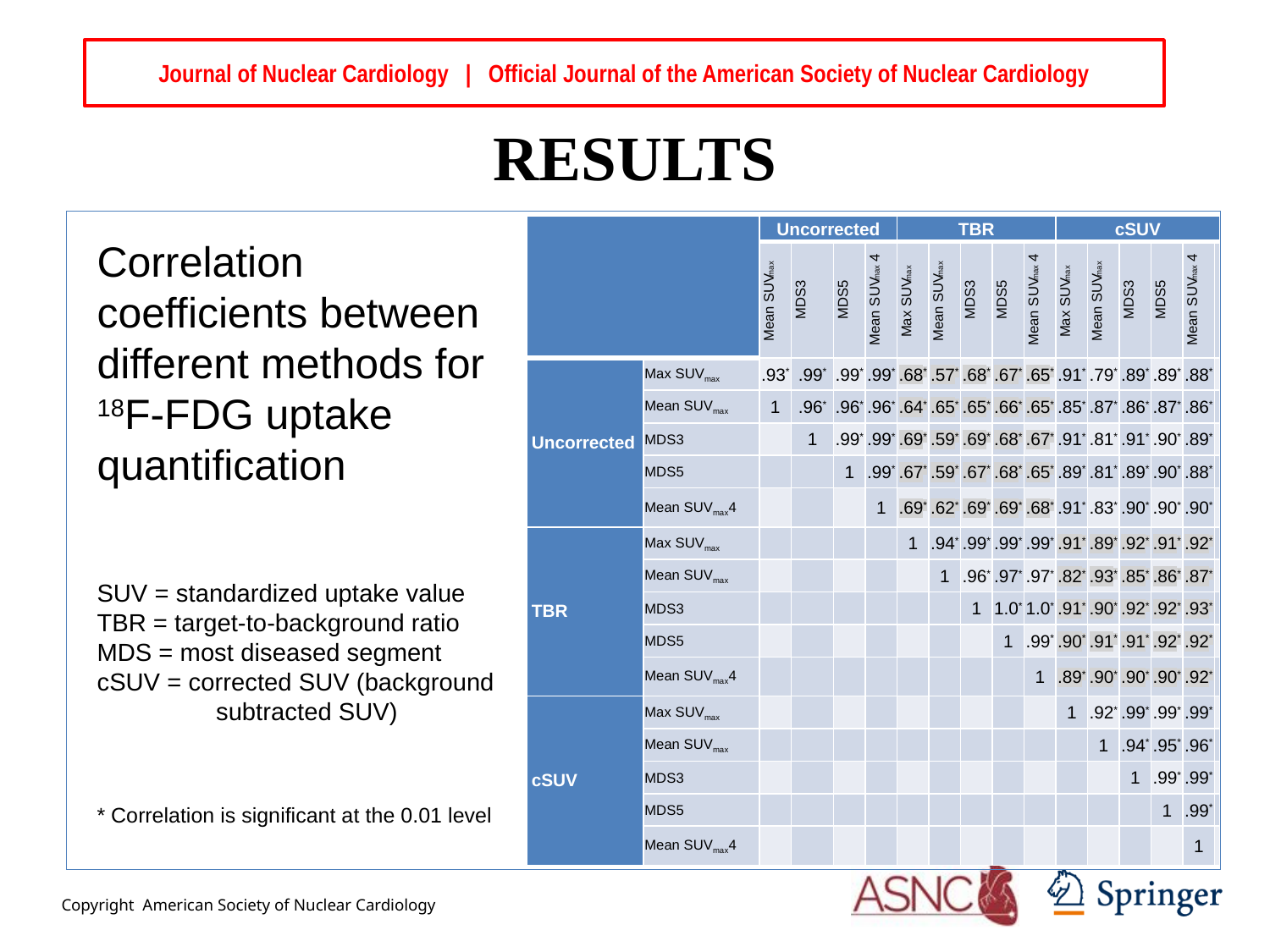

Journal of Nuclear Cardiology | Official Journal of the American Society of Nuclear Cardiology
# RESULTS
| | | Uncorrected | | | | TBR | | | | | cSUV | | | | | |
| --- | --- | --- | --- | --- | --- | --- | --- | --- | --- | --- | --- | --- | --- | --- | --- | --- |
| | | Mean SUVmax | MDS3 | MDS5 | Mean SUVmax4 | Max SUVmax | Mean SUVmax | MDS3 | MDS5 | Mean SUVmax4 | Max SUVmax | Mean SUVmax | MDS3 | MDS5 | Mean SUVmax4 | |
| Uncorrected | Max SUVmax | .93\* | .99\* | .99\* | .99\* | .68\* | .57\* | .68\* | .67\* | .65\* | .91\* | .79\* | .89\* | .89\* | .88\* | |
| | Mean SUVmax | 1 | .96\* | .96\* | .96\* | .64\* | .65\* | .65\* | .66\* | .65\* | .85\* | .87\* | .86\* | .87\* | .86\* | |
| | MDS3 | | 1 | .99\* | .99\* | .69\* | .59\* | .69\* | .68\* | .67\* | .91\* | .81\* | .91\* | .90\* | .89\* | |
| | MDS5 | | | 1 | .99\* | .67\* | .59\* | .67\* | .68\* | .65\* | .89\* | .81\* | .89\* | .90\* | .88\* | |
| | Mean SUVmax4 | | | | 1 | .69\* | .62\* | .69\* | .69\* | .68\* | .91\* | .83\* | .90\* | .90\* | .90\* | |
| TBR | Max SUVmax | | | | | 1 | .94\* | .99\* | .99\* | .99\* | .91\* | .89\* | .92\* | .91\* | .92\* | |
| | Mean SUVmax | | | | | | 1 | .96\* | .97\* | .97\* | .82\* | .93\* | .85\* | .86\* | .87\* | |
| | MDS3 | | | | | | | 1 | 1.0\* | 1.0\* | .91\* | .90\* | .92\* | .92\* | .93\* | |
| | MDS5 | | | | | | | | 1 | .99\* | .90\* | .91\* | .91\* | .92\* | .92\* | |
| | Mean SUVmax4 | | | | | | | | | 1 | .89\* | .90\* | .90\* | .90\* | .92\* | |
| cSUV | Max SUVmax | | | | | | | | | | 1 | .92\* | .99\* | .99\* | .99\* | |
| | Mean SUVmax | | | | | | | | | | | 1 | .94\* | .95\* | .96\* | |
| | MDS3 | | | | | | | | | | | | 1 | .99\* | .99\* | |
| | MDS5 | | | | | | | | | | | | | 1 | .99\* | |
| | Mean SUVmax4 | | | | | | | | | | | | | | 1 | |
Correlation coefficients between different methods for 18F-FDG uptake quantification
SUV = standardized uptake value
TBR = target-to-background ratio
MDS = most diseased segment
cSUV = corrected SUV (background
 subtracted SUV)
* Correlation is significant at the 0.01 level
Copyright American Society of Nuclear Cardiology

## Slide 5
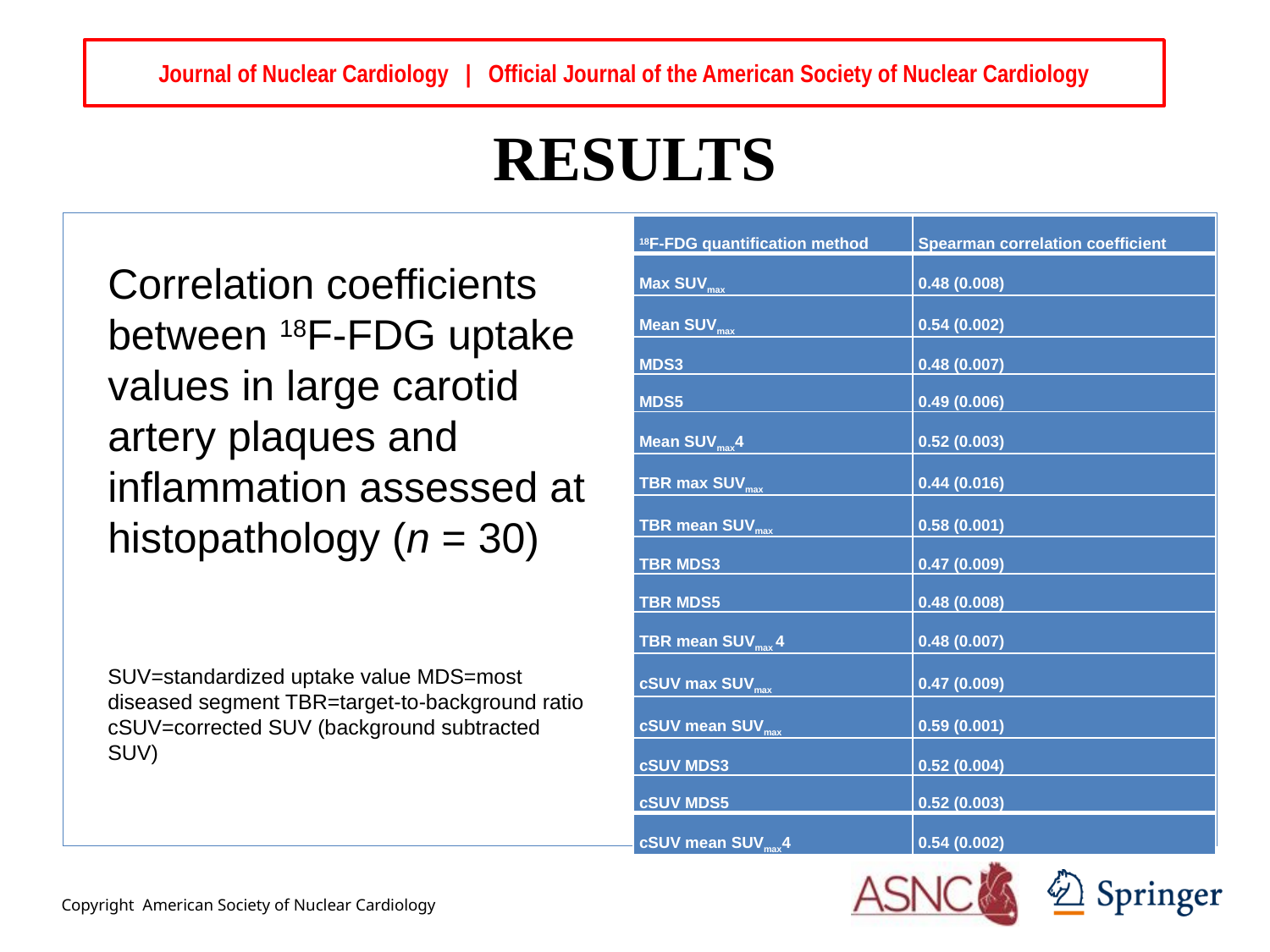

Journal of Nuclear Cardiology | Official Journal of the American Society of Nuclear Cardiology
# RESULTS
| 18F-FDG quantification method | Spearman correlation coefficient |
| --- | --- |
| Max SUVmax | 0.48 (0.008) |
| Mean SUVmax | 0.54 (0.002) |
| MDS3 | 0.48 (0.007) |
| MDS5 | 0.49 (0.006) |
| Mean SUVmax4 | 0.52 (0.003) |
| TBR max SUVmax | 0.44 (0.016) |
| TBR mean SUVmax | 0.58 (0.001) |
| TBR MDS3 | 0.47 (0.009) |
| TBR MDS5 | 0.48 (0.008) |
| TBR mean SUVmax 4 | 0.48 (0.007) |
| cSUV max SUVmax | 0.47 (0.009) |
| cSUV mean SUVmax | 0.59 (0.001) |
| cSUV MDS3 | 0.52 (0.004) |
| cSUV MDS5 | 0.52 (0.003) |
| cSUV mean SUVmax4 | 0.54 (0.002) |
Correlation coefficients between 18F-FDG uptake values in large carotid artery plaques and inflammation assessed at histopathology (n = 30)
SUV=standardized uptake value MDS=most diseased segment TBR=target-to-background ratio cSUV=corrected SUV (background subtracted SUV)
Copyright American Society of Nuclear Cardiology

## Slide 6
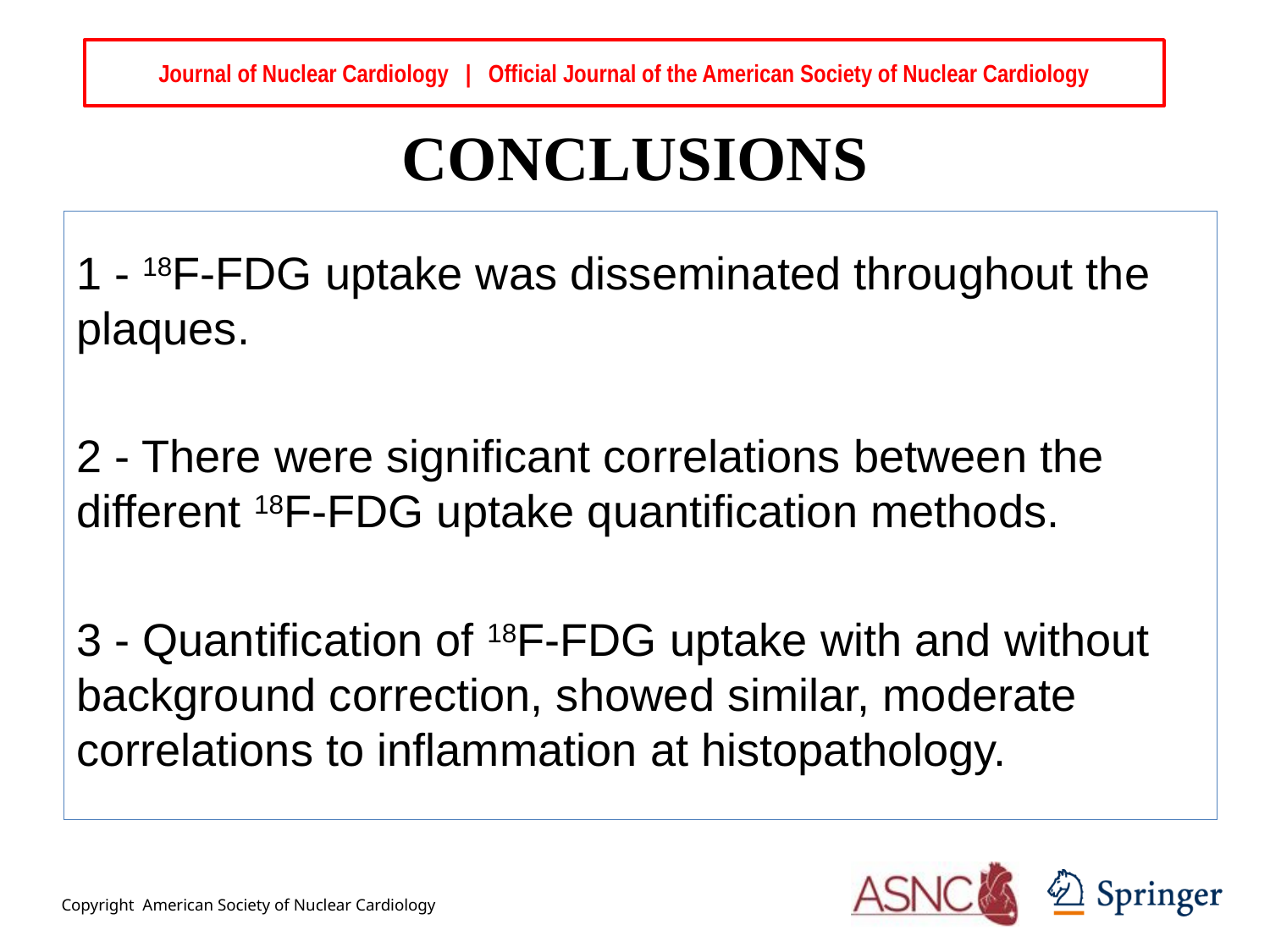

Journal of Nuclear Cardiology | Official Journal of the American Society of Nuclear Cardiology
# CONCLUSIONS
1 - 18F-FDG uptake was disseminated throughout the plaques.
2 - There were significant correlations between the different 18F-FDG uptake quantification methods.
3 - Quantification of 18F-FDG uptake with and without background correction, showed similar, moderate correlations to inflammation at histopathology.
Copyright American Society of Nuclear Cardiology
